# Commentary: Ventriculo-arterial (un)coupling in septic shock: impact of current and upcoming hemodynamic drugs

**DOI:** 10.3389/fcvm.2023.1320213

**Published:** 2023-12-14

**Authors:** Xiaoyang Zhou, Zhaojun Xu, Yuyi Sha

**Affiliations:** Department of Intensive Care Medicine, Ningbo No.2 Hospital, Ningbo, Zhejiang, China

**Keywords:** ventriculo-arterial coupling, cardiovascular, septic shock, fluid responsiveness, hemodynamic drug

A Commentary on Ventriculo-arterial (un)coupling in septic shock: impact of current and upcoming hemodynamic drugs By Zhou X, Xu Z and Sha Y. (2023) Front. Cardiovasc. Med. 5:1172703. doi: 10.3389/fcvm.2023.1172703

## Introduction

In a recent release of the journal, Demailly et al. conducted a mini review on the relationship between hemodynamic drugs and ventriculo-arterial coupling (VAC) in septic shock (1). We have read this review with great interest and benefit greatly from its profound insights. We congratulate the authors for their excellent work on the comprehensive evaluation concerning the hemodynamic impacts of multiple interventions (mainly vasoactive drugs, inotropic agents, and *β*-blockers) on the VAC. We want, however, to add a few general comments regarding the impact of fluid therapy on the VAC, which is not mentioned in the mini review.

## Discussion

In recent years, the concept of VAC has gained popularity in the field of critical care medicine. The VAC describes an interdependent and interactive work manner between the ventricles and the large arteries and determines the cardiovascular work efficiency (2). The VAC index, defined as the ratio of effective arterial elastance (Ea) to left ventricular end-systolic elastance (Ees), integrates cardiac contractility, arterial load and the ventricular-arterial interactions in a common framework. Thus, during the resuscitation of septic shock, any treatment measures that affect these components will potentially alter the cardiovascular profile and finally impact the VAC. In addition to vasoactive and inotropic drugs, fluid is also a fundamental hemodynamic drug for the treatment of circulatory shock. Clinically, fluid expansion attempts to increase cardiac output by restoring cardiac preload and finally improve tissue perfusion. However, fluid-induced preload increases may cause different cardiovascular responses, including varied changes in Ea and Ees, and exhibit complex effects on the VAC, which is difficult to determine and may depend on fluid responsiveness.

The Ees is a parameter of cardiac contractility and represents the slope of left ventricular end-systolic pressure-volume relationships (ESPVR), of what the theoretical framework arises from the time-varying elastance model of the left ventricle (2). Several decades ago, a series of experiments on isolated canine ventricles indicated that the slope of the ESPVR (i.e., Ees) under various volume conditions was approximately linear over a physiological range and was insensitive to volume changes (3, 4). Several clinical studies further confirmed the preload-independence of Ees (5, 6). Huette et al. (5) conducted a prospective study on cardiothoracic surgery patients to evaluate the effect of fluid challenge on the VAC. Their findings indicated that fluid challenge had no significant effect on the Ees, whether or not the patients were volume-responsive. Similarly, the study by Li et al. (6) also demonstrated that the Ees is independent of volume changes. They found no or tiny impact of fluid challenge on the Ees in patients with septic shock, regardless of fluid responsiveness. However, a recent study observed a significant increase in the Ees after fluid expansion in septic patients (7). Unfortunately, the positive results found in this study (7) should be interpreted with caution because fluid expansion might be administrated concomitantly with norepinephrine or dobutamine due to the long duration of fluid administration (3 h). Of note, both norepinephrine and dobutamine have been validated to remarkably enhance the Ees (1, 8).

With respect to the relationship between fluid therapy and the Ea, the current evidence consistently suggested that fluid expansion significantly reduced the Ea in fluid-responsive patients (5–7). It is not surprising because fluid-responsive patients typically have insufficient blood volume and probably have higher levels of sympathetic activation to maintain tissue perfusion than non-responders (5, 9). Thus, once the blood volume is restored by fluid expansion, sympathetic activation would be decreased, leading to a reduction in vascular tone and Ea. Conversely, fluid expansion seems can induce a trend toward an increase of Ea in preload-independent patients, as indicated by the studies of Huette et al. (5) and Li et al. (6). Theoretically, further administrating fluid to preload-independent patients potentially prompts fluid overload and congestion, which are well-known clinical conditions characterized by sympathetic stress and renin-angiotensin system activation, thereby resulting in an increased trend of Ea. Moreover, two studies reversely verified the negative impact of fluid expansion on the Ea in this cohort population: decongestive therapy using diuretics reduced the Ea and improved the VAC in congestive patients (10, 11). Accordingly, the fluid-induced changes in the Ea, rather than the Ees, seem to be the primary determinant of fluid-induced VAC changes.

## Conclusion

Overall, the ventricular-arterial system is interconnected and should not considered as two isolated structures. Any intervention affecting components of the cardiovascular system would produce complicated impacts on the VAC. The comprehensive effect of fluid infusion on the interaction between cardiac and arterial performance is difficult to determine and may depend on the volume status.
